# Exosomal Proteins and miRNAs as Mediators of Amyotrophic Lateral Sclerosis

**DOI:** 10.3389/fcell.2021.718803

**Published:** 2021-09-10

**Authors:** Qiao Yi Chen, Ting Wen, Peng Wu, Rui Jia, Ronghua Zhang, Jingxia Dang

**Affiliations:** ^1^Department of Cell Biology and Genetics, School of Basic Medical Sciences, Xi’an Jiaotong University, Xi’an, China; ^2^Department of Neurology, The First Affiliated Hospital of Xi’an Jiaotong University, Xi’an, China

**Keywords:** exosomes, ALS, protein misfolding, miRNA, nanotechnology

## Abstract

Recent advances in the neurobiology and neurogenerative diseases have attracted growing interest in exosomes and their ability to carry and propagate active biomolecules as a means to reprogram recipient cells. Alterations in exosomal protein content and nucleic acid profiles found in human biological fluids have been correlated with various diseases including amyotrophic lateral sclerosis (ALS). In ALS pathogenesis, these lipid-bound nanoscale vesicles have emerged as valuable candidates for diagnostic biomarkers. Moreover, their capacity to spread misfolded proteins and functional non-coding RNAs to interconnected neuronal cells make them putative mediators for the progressive motor degeneration found remarkably apparent in ALS. This review outlines current knowledge concerning the biogenesis, heterogeneity, and function of exosomes in the brain as well as a comprehensive probe of currently available literature on ALS-related exosomal proteins and microRNAs. Lastly, with the rapid development of employing nanoparticles for drug delivery, we explore the therapeutic potentials of exosomes as well as underlying limitations in current isolation and detection methodologies.

## Introduction

Neurodegenerative diseases (ND), including Alzheimer’s disease (AD), Parkinson’s disease (PD), Huntington’s disease, prion disease, and amyotrophic lateral sclerosis (ALS), are characterized by progressive nervous system dysfunction and neuronal loss (Martin, 1999; Quek and Hill, 2017). This review centers around ALS, which is a fatal neurodegenerative disease characterized by motor neuron (MN) degeneration and progressive muscle weakness, and in most cases, will lead to respiratory failure within 3 to 5 years of disease onset (Cleveland and Rothstein, 2001; Chiò et al., 2009; Brown and Al-Chalabi, 2017; Hosaka et al., 2019; Roy et al., 2019). Unfortunately, molecular mechanisms underlying motor neuron degeneration remain elusive, creating tremendous challenges for finding reliable diagnostic biomarkers and treatment strategies, though various hypotheses have been proposed, including both genetic and non-genetic causes. Currently, roughly 10% of familial ALS (fALS) cases have been linked to inherited gene mutations, including superoxide dismutase 1 (SOD1), hexanucleotide repeats in *C9orf72*, and TAR DNA binding protein 43 (TDP-43), while 90% of cases have no family history, also known as sporadic ALS (sALS) (Chia et al., 2018; Kim et al., 2020; Van Harten et al., 2021). In both fALS and sALS, abnormal deposition of misfolded proteins has been implicated as a prominent pathological hallmark. In recent years, extracellular vesicles (EVs) such as exosomes have been implicated as potential vehicles for misfolded proteins and non-coding RNAs, and are likely responsible for the apparent spread of interconnected neuronal cell death found in ALS pathogenesis (Coleman and Hill, 2015; Wu et al., 2016; Gagliardi et al., 2020).

Extracellular vesicles are lipid bound nanoscale spheroid structures secreted by cells into the extracellular space (Doyle and Wang, 2019; Liu et al., 2019; Turchinovich et al., 2019). These tiny vesicles were first discovered in the late 1960s when researchers observed the secretion of ∼100 nm vesicles from the plasma membrane of chondrocytes and platelets (Bonucci, 1967; Wolf, 1967; Anderson, 1969; Pegtel and Gould, 2019). Since then, studies stemming from these foundational findings have led to newly defined classifications and functions of EVs, which were long thought to merely expel waste products from cells. Rather, it has become increasingly evident that EVs are released by almost all cell types, found highly abundant in biological fluids, such as blood, cerebrospinal fluid (CSF), saliva, and urine, and act as efficient transporters of bioactive molecules such as proteins, lipids, RNAs, and DNA fragments between cells, thus playing a vital role in cell-cell communication (Bard et al., 2004; Hegmans et al., 2004; Valadi et al., 2007; Balaj et al., 2011; Llorente et al., 2013; Kahlert et al., 2014; Thakur et al., 2014; Abels and Breakefield, 2016; Skotland et al., 2017; van Niel et al., 2018; Liu et al., 2019; Turchinovich et al., 2019; Zhou et al., 2020). In 1981, Trams et al. reported two distinct types of vesicles secreted by mammalian cells, ones that were ∼40 nm in diameter and require a high *g* force for sedimentation (10,000 × *g* for up to 120 min), and ones that were >500 nm in diameter, which required a lower *g* force (10,000 × *g* for a∼30 min) (Trams et al., 1981; Pegtel and Gould, 2019). Today, based on their size, origin, biogenesis, content, and function, EVs are classified into three main types: apoptotic bodies (ABs), microvesicles (MVs), and exosomes. ABs are by-products of apoptosis with an average diameter of 1–5 μm, whereas MVs are formed by outward budding of the plasma membrane and range between 0.1–2 μm in diameter. Exosomes are the smallest type of EVs, usually between 30–150 nm in size (Shao et al., 2018). Recently, exosomes have been further divided into new subpopulations known as large exosome vesicles (Exo-L, 90–120 nm), small exosome vesicles (Exo-S, 60–80 nm), and non-membranous nanoparticles called exomeres (∼35 nm) (Zhang et al., 2018; Zhou et al., 2020).

## Exosome Biogenesis and Integration

xosomes, originally known as shedding vesicles, are influenced by the cell microenvironment and secreted by certain stimuli (Liu et al., 2019). Biogenesis of exosomes occurs through both endosomal pathway as well as budding from the plasma membrane (Fang et al., 2007; Pegtel and Gould, 2019; Zhou et al., 2020). The endosomal biogenesis hypothesis emerged from evidence on TfR secretion by reticulocytes (Pan and Johnstone, 1983; Harding et al., 1984). In this mode of biogenesis, as evidenced by both electron microscopy and genetic studies, proteins, lipids, and nucleic acids are first endocytosed into early endosomes, from which they mature into late endosomes known as multi-vesticular bodies (MVBs), and later released into the extracellular space as exosomes through fusion with lysosomes or the plasma membrane (Abels and Breakefield, 2016; Shao et al., 2018; Liu et al., 2019; Shaimardanova et al., 2020). During this vesicular transporting process, Rab27a and Rab27b proteins and their effectors Munc13-4, Slp4, and Slac2b have been shown to help direct and transfer the vesicles to dock at the plasma membrane (Zerial and McBride, 2001). Rab proteins are the largest family of small monomeric GTPases, and mainly function to coordinate vesicle motility, formation, and transport through interaction with microtubules and actin filaments. In addition, Rab proteins can act as an on/off switch by oscillating regularly from GTP (active) to GDP-bound (non-active) states, which create the temporal regulation necessary for localized membrane transport (Zerial and McBride, 2001). To underscore its importance, it is reported that in certain cell lines, loss of Rab27 function alone can lead to a 50 to 75% reduction in exosome formation. In addition, the less reported mode of exosome biogenesis occurs through direct budding from the plasma membrane, and there is ample evidence from electron microscopy experiments demonstrating that exosomes can bud from the plasma membrane of various mammalian cells as well as *C. elegans* (Anderson et al., 2005; Shen et al., 2011; Wehman et al., 2011; Bianchi et al., 2014). Interestingly, exosomes containing CD9 and CD63 proteins selectively bud from the plasma membrane, which suggests that exosome composition may influence the elected pathway of biogenesis, although further investigation is warranted to answer this question.

Understanding how proteins are targeted and trafficked into exosomes is a central part of exosome biogenesis. In typical organelle biogenesis, short peptide signals serve as recognition sites for import receptors. Meanwhile, the pathway of exosome secretion is regulated by either the endosomal sorting complex required for transport (ESCRT)-dependent or ESCRT-independent pathways. ESCRT has been evidenced to facilitate various biological processes including plasma membrane repair, nuclear envelope sealing, as well as virus budding through catalyzing membrane scission (Radulovic and Stenmark, 2018). In exosome biogenesis, the ESCRT machinery, which consists of four proteins (ESRT-0, ESRT-I, ESRT-II, and ESRT-III) and three accessory proteins (ALIX complex, VTA1, and VPS4), functions to sort ubiquitinylated proteins into intraluminal vesicles (ILVs) (Gill et al., 2007). On the other hand, ESCRT-independent exosome formation depends on either ceramide formation or tetraspanins such as CD9, CD63, CD81, and CD82 for protein sorting rather than ubiquitylation (Trajkovic et al., 2008). Noted, inhibition of the enzyme responsible for generating ceramide can directly reduce exosome biogenesis due to lack of membrane curvature support needed for membrane vesiculation. In addition, low ceramide levels can also indirectly impact exosome biogenesis through regulation of cellular apoptosis, metabolism, and autophagy (Trajkovic et al., 2008; Ogretmen, 2018). Interesting to note, cancer cells that lack the autophagy-related 5 (Atg5) protein show a significant reduction in exosome production (Guo et al., 2017). Conversely, knockout of Atg5 in neuronal cells via CRISPR/Cas9 can lead to an increase in exosome production, suggesting the dual-effect of autophagy on exosome secretion (Abdulrahman et al., 2018).

Exosome uptake by recipient cells depends on the interaction between proteins on the surface of exosomes as well as the recipient cells. Transmembrane proteins on the surface of exosomes can function as signaling molecules and deliver functional receptors and signaling pathways to recipient cells (Kalluri and LeBleu, 2020). Several reports have suggested that specific ligand-receptor interactions play a key role in exosome-cell binding and uptake (Pegtel and Gould, 2019). These include direct and indirect binding of exosomes to cells based on expression of PS receptors, lectins, glycans, integrins, and other cell adhesion molecules. Other exosomal surface proteins, such as CD9 and CD81, have also been implicated in exosome-cell fusion events, though there is no direct evidence that they mediate exosome-cell fusion. In most studies, the stromal cell recipients of cancer cell-derived exosomes are cancer-associated fibroblasts (CAFs) and immune cells. Exosome uptake by recipient cells is also related to multiple mechanisms, including micropinocytosis, phagocytosis, clathrin-dependent endocytosis, and clathrin-independent endocytosis (Zhang and Yu, 2019). For example, exosomes expressing the Tspan8-alpha4 complex are most easily taken up by endothelial and pancreatic cells, and CD54 is the major ligand (Nazarenko et al., 2010; Rana et al., 2012). Zomer et al. (2015) used the Cre-LoxP system to demonstrate that EVs carrying mRNAs involved in migration and metastasis released by malignant tumor cells can be taken up by less malignant tumor cells located within the same and distant tumors. In human pancreatic cancer cells, integrin CD47 expression has been shown to promote exosome uptake by micropinocytosis, and human melanoma cells can take up exosomes through fusion with the cell membrane, especially when the tumor microenvironment is acidic (Parolini et al., 2009; Kamerkar et al., 2017). In the central nervous system (CNS), neurosecretory PC12 cell-derived exosomes rely on clathrin-dependent endocytosis and macropinocytosis for uptake (Tian et al., 2014).

## Exosome Function and Heterogeneity in the Brain

Exosomes were originally thought to only participate in protein quality control through the removal of waste proteins and macromolecules from the plasma membrane as evidenced during the maturation process of sheep reticulocytes (Harding and Stahl, 1983; Pan and Johnstone, 1983; Johnstone et al., 1987; Quek and Hill, 2017). Interestingly, in amoeboid cells, since exosomal proteins are sorted and enriched at the posterior pole, polarity is created to facilitate cell migration (Shen et al., 2011). Exosomes can also be integrated into the extracellular matrix (ECM) and exert influence both on its composition and function, such as promoting growth and spread of plaques and amyloid aggregates through modulation of ECM in neurodegenerative disorders (Fevrier et al., 2004; Quek and Hill, 2017; Cheng et al., 2018). Current understanding and research on exosome function center around their ability to transport signals and contents between cells thereby facilitating inter-cellular communication and contributing to cell maintenance, tumor progression, as well as promoting the onset and progression of neurodegenerative diseases. Specifically, exosomes can either travel through the blood circulation system or act via paracrine and autocrine signaling to reach distant cells and promote cellular communication through endocytosis, membrane fusion, or receptor-ligand binding. Exosomes have a phospholipid bilayer that protects encapsulated cargo from being degraded during the process of being transported to targeted adjacent or distant cells. Exosomes carry a range of highly functional contents including nucleic acids, transcription factors, proteins and lipids that can exert transformational effects in targeted cells (Alonso and Millán, 2001; Heinzelman et al., 2009; Chow et al., 2014; Melo et al., 2014; Kawikova and Askenase, 2015). For example, tumor cell-derived exosomes have been shown to stimulate cell proliferation and angiogenesis (Turturici et al., 2014; Gagliardi et al., 2020). In addition, exosomes have also been implicated to influence immunosuppression through mediating PD-L1 signaling, transferring infectious prions, as well as delivering functional miRNA content to exert epigenetic modifications and gene expression changes in recipient cells (Cheng et al., 2018; Saeedi et al., 2019). Exosomes are capable of crossing the blood brain barrier (BBB), and thus can transport disease-related proteins and molecules to and from CNS cells. In the CNS, exosomes have been found to mediate the secretion of various neurodegenerative-related proteins including amyloid beta (Aβ), PrP, tau, and SOD1, and stimulate myelin formation, synaptic function, plasticity, neuronal cell survival and repair (Fevrier et al., 2004; Fauré et al., 2006; Krämer-Albers et al., 2007; Bakhti et al., 2011; Lachenal et al., 2011; Verweij et al., 2011, 2015; Wang et al., 2011; Hurwitz et al., 2017; Quek and Hill, 2017; Doyle and Wang, 2019). Currently, neurodegeneration of ALS has only been evidenced to originate from motor neurons. But considering the ability of exosomes to across the BBB, it would be interesting to investigate whether the source of ALS-related disease biomolecules comes from elsewhere in the body. For example, in Parkinson’s disease, origin of neurodegeneration has been traced back to the appendix which is rich in aggregated α-synuclein (α-syn) (major component of Lewy bodies). α-syn can be propagated between neurons and travel from gut to brain via the vagal nerve (Killinger et al., 2018). The gut-brain axis has also been implicated in ALS, including the potential effect of gut microbiome on disease severity. However, the underlying mechanisms are unclear, and future studies may consider looking into potential disease protein propagation via exosomes from other parts of the body such as the gut. Overall, exosomes have multifaceted functions that enable their contribution to a wide range of physiological processes including cellular homeostasis, metabolic regulation, aging, as well as the spread of infectious and non-infectious diseases.

While there is limited understanding on mechanisms driving the highly variable functions of exosomes, evidence suggests that exosome heterogeneity stems from differences in their origin, composition, size, and function. Exosomes are naturally secreted by both animals and plants, which are found similar in structure. Mammalian exosomes can derive from almost all cell types including mesenchymal stem cells (MSCs) and CNS cells (Potolicchio et al., 2005; Verkhratsky et al., 2012; Frühbeis et al., 2013; Delpech et al., 2019). Cellular origin and size can greatly impact the amount and type of exosomal content, which consists of proteins from the membrane, nucleus, and cytosol, as well as DNA, mRNA, and non-coding RNA species (van Balkom et al., 2015; Keerthikumar et al., 2016; Lasda and Parker, 2016; Pathan et al., 2019; Kalluri and LeBleu, 2020). Since exosomes inherit their biomolecules from parent cells, much of their heterogeneity in function will come from their cell of origin. CNS cells including neurons, oligodendrocytes, microglia, and astrocytes, are all known to secrete exosomes that are distinct in function. For example, secretion of cortical neuron-derived exosomes is stimulated by potassium-induced depolarization, and they characteristically shuttle adhesion molecules including NCAM1, NCAM2, and L1, synaptotagmin 4 (Syt4), miR-124a, as well as mRNAs associated with the activity-regulated-cytoskeleton-associated protein (Arc) (Watanabe et al., 2014; Pastuzyn et al., 2018; Riva et al., 2019). In oligodendrocytes, exosomes are secreted as a result of glutamate-induced Ca^2+^ influx and subsequent activation of small GTPase Rab35, and carry various enzymes such as SOD1, oxidative stress alleviating peroxiredoxins (PRDX-1,2), and dihydropyrimidinase-related proteins (DPYL-2,3), and function in neuronal protection under hypoglycemic and hypoxic conditions (Krämer-Albers et al., 2007). Microglia-derived exosomes may play a role in neuroimmune functions as they carry similar proteins as exosomes from B cells and dendritic cells, including CD13, monocarboxylate transporter (MCT1), and proinflammatory cytokine IL-1β (Riva et al., 2019). In addition, exosomes derived from astrocytes are released after increased intracellular Ca^2+^, heat or oxidative stress conditions, and often contain heat shock proteins (HSP70), PAR4, and ceramide, which have been linked to neurodegenerative diseases such as ALS (Verkhratsky et al., 2012).

Pathogenesis of neurodegenerative diseases have been strongly linked to the abnormal deposition of aggregate and misfolded proteins, with growing evidence pointing to the prion-like role of exosomes in propagating disease protein transmission. For example, exosomes have been evidenced to carry toxic forms of α-synuclein (α-syn) in PD, tau and Aβ in AD, and SOD1 in ALS (Aguzzi and Rajendran, 2009; Vingtdeux et al., 2012; Schneider and Simons, 2013; Joshi et al., 2015). However, some have argued against this hypothesis since exosomes can be effective vehicles for removing toxic proteins such as synaptotoxic Aβ species through the endosomal pathway and aid neuron cell protection (Bobrie et al., 2012; Yuyama et al., 2015). While early functional hypotheses regard exosomes as garbage bags that serve to expel unwanted components from the cell, it is unknown whether these constituents are degraded along with or excreted from the exosomes that contain them. Current literature suggests that low exosome levels can have damaging effects. For example, reduction in exosome levels in the CSF is correlated with Aβ accumulation in the brains of aged mice (Yuyama et al., 2015). In addition, low exosome levels can lead to cytoplasmic DNA accumulation in the cytoplasm and promote ROS-dependent DNA damage and cell death (Takahashi et al., 2017). Potential causes for reduced exosome levels include endocytic disruption mediated by endosome enlargement and dynein dysfunction, can disrupt normal endosome trafficking (Kimura et al., 2009, 2014). Current evidence indicates that exosomes can serve as both transfer and excretion vehicles for molecules. And their contribution to disease progression, either in positive or negative ways, may be highly dependent on the type of the protein or nucleic acids encapsulated. Thus, in effort to deepen our understanding of exosomal function, it is important to acknowledge that exosome is a broad term and that different subclasses of exosomes can have vastly different functions. In addition to size, content, and tissue of origin, exosomes can also be categorized based on distinct biochemical and biophysical properties (Willms et al., 2018; Wang et al., 2019; Singh et al., 2021). Various methods have been explored in extracting tissue specific exosomes. For example, proximity-dependent barcoding assay has been developed to identify distinct surface proteins on exosomes through NGS and antibody-DNA conjugates (Wu et al., 2019). These surface proteins provide important evidence for tissue of origin. In addition, through sucrose gradient density ultracentrifugation, Willms et al. (2016) identified two subpopulations of exosomes across various cell lines and plasma, including A431 human squamous carcinoma, melanoma, H5V mouse heart endothelial, N2a mouse neuroblastoma, MSC hTERT immortalized mesenchymal stem cells. Findings from this study revealed that cells can secrete two major subpopulations of exosomes: lower (LD) and higher density (HD) fractions, each with distinct size, RNA, and protein composition. Moreover, LD and HD exosomes can elicit differential gene expression changes in recipient cells. A potential explanation for exosome heterogeneity is their biogenesis pathways, and that impairment of one pathway may yield increased secretion of a certain subclass of exosomes. Thus, understanding the functional differences between different subclasses of exosomes and the mechanisms of biogenesis are crucial for exosome-dependent diagnosis and therapeutics. Nonetheless, because of their unanimous presence in circulating body fluids and the ability to carry abnormal protein and nucleic acid contents in neurodegenerative disease patients, exosomes have the potential to serve as non-invasive biomarkers. In addition, since exosomes can cross the BBB, they are likely candidates for shuttling siRNAs and pharmacological molecules into the extracellular space or target cells and act as vectors for ND therapeutics.

## Excretion and Propagation of ALS-Related Proteins Through Exosomes

**FIGURE 1:**
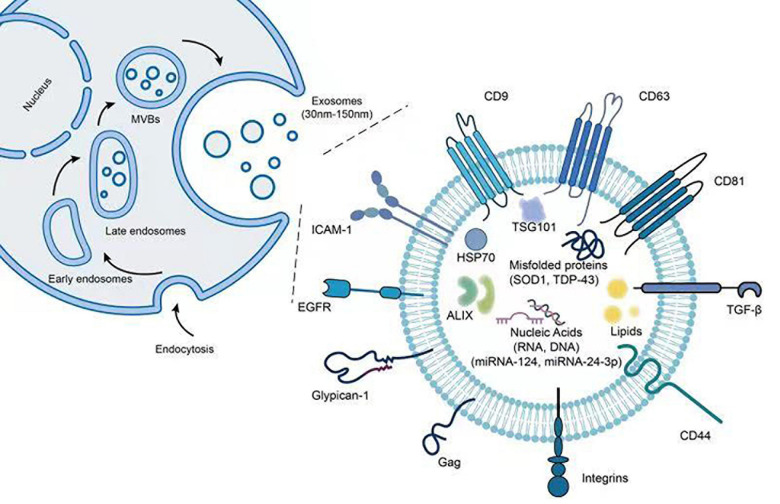
Illustration of exosome structure and associated proteins and miRNAs. The figure demonstrates the steps of exosome biogenesis as well as potential biomolecules found both inside as well as on the surface of exosomes.

Exosomes are known to contain and express various types of proteins including inner and outer membrane proteins such as CD63 and CD81, cell adhesion proteins including tetraspanins and integrins, heat shock proteins such as Hsp70, peripheral surface proteins such as wingless (wnt) proteins and first apoptosis signal ligand, as well as lipases, proteases, metabolic, and RNA editing enzymes (Stoorvogel et al., 2002; Février and Raposo, 2004; Kim et al., 2007; Gross and Zelarayán, 2018). Notably, exosomes from single cell lines have been shown to carry more than 3,000 proteins (Li et al., 2016). Figure 1 provides an illustration for common biomolecules found both on the surface and the inside of exosomes. Apart from commonly found proteins such as tubulin, actin, and glycolytic enzymes, exosomes can also carry misfolded proteins to recipient cells and stimulate the onset and progression of neurodegenerative disorders (Peng et al., 2020). In fact, deposition and aggregation of misfolded proteins in brain tissues are hallmark features for many neurodegenerative diseases. Recently, exosomes have been evidenced to package and transport key proteins involved in the onset and spread of ALS, including SOD1, TDP-43, dipeptide-repeat proteins (DPRs), and fused in sarcoma (FUS) (Grad et al., 2014; Silverman et al., 2016, 2019; Gagliardi et al., 2020; Wang and Zhang, 2020).

*In vitro* models for ALS studies often employ mouse motor neuron-like NSC-34 cells. A study led by Gomes et al. (2007) first showed that NSC-34 cells either overexpressing wild-type (WT) or mutant human SOD1, can both secrete SOD1 via exosomes. SOD1 in its WT form, is a cytosolic and mitochondrial enzyme known for its neuronal protective function against reactive oxygen intermediates such as superoxide molecules (Clement et al., 2003). Under normal circumstances, mutant forms of SOD1 are also present in exosomes, although in lesser extent compared to WT SOD1. While WT SOD1 can suppress microglial activation, mutant SOD1 has been linked to neuronal inflammation, microgliosis, and neuron death (Urushitani et al., 2006). Apart from mutant SOD1, misfolded SOD1 proteins, either in mutant or WT forms, have also been implicated in ALS. In NSC-34 cells, Grad et al. (2014) reported that misfolded mutant and WT SOD1 proteins can move between cells through both exosomes and protein aggregates. This has also been demonstrated previously by Basso et al., where the researchers demonstrated that astrocyte-derived exosomes can efficiently cargo misfolded SOD1 protein to neighboring spinal neurons to promote cell death (Basso et al., 2013). Moreover, misfolding of WT SOD1 propagation is a sustained phenomenon, as evidenced by HEK293 cells cultured in conditioned media (Grad et al., 2014). Silverman et al. (2019) reported that EVs from mouse brain and spinal cord tissues as well as human SOD1 fALS patient spinal cord tissues contained high levels of misfolded as well as non-native disulfide-cross-liked SOD1 aggregates. In addition, the team showed that brain astrocytes and neurons, but not microglia, were the main source of EVs (Silverman et al., 2019). Conversely, Massenzio et al. (2018) demonstrated that microglial cells were capable of releasing SOD1 via exosomes. The study also showed that while intercellular accumulation of mutant SOD1 led to microglial activation and autophagy dysfunction, induction of autophagy through trehalose treatment significantly inhibited SOD1 accumulation and neurotoxicity through proteasome degradation (Massenzio et al., 2018). Overall, these studies help to support the prion-like transmission hypothesis that propagated protein misfolding may be responsible for the spatiotemporal progression of ALS (Ravits and La Spada, 2009; Polymenidou and Cleveland, 2011).

TAR DNA binding protein 43 is an RNA/DNA binding protein that belongs to the heterogenous nuclear ribonucleoprotein (hnRNP) family and is highly involved in regulating mRNAs for neuron development. TDP-43 inclusion, potentially driven by hyper-phosphorylation and ubiquitination, is the most prominent histopathological feature in sALS, which reflects its prominent role in ALS pathology (Neumann et al., 2006; Feneberg et al., 2014). In addition, exosomal TDP-43 ratio is positively correlated with ALS disease progression, suggesting its potential to act as a biomarker for ALS diagnosis and rate of progression (Chen et al., 2020). Interestingly, TDP-43 pathology can also be found in fALS, though only in cases that lack SOD1 mutation, suggesting a competing or differential disease mechanism between the two. Aggregates of TDP-43 in ALS patients have been evidenced to be released by exosomes, and are characterized by their granular appearance in the cytoplasm found throughout the brain, including spinal cord, cortex, thalamus, and basal ganglia. Nonaka et al. (2013) reported exosomes from ALS brain tissues can promote TDP-43 aggregation in human neuroblastoma SH-SY5Y cells, indicating strong prion-like properties and the critical role of exosomes for transporting TDP-43 aggregates. Another study conducted by Iguchi et al. (2016) reported high levels of exosomal TDP-43 in ALS patients, and that the secretion and propagation of TDP-43 via exosomes in Neuro2a cells are in part driven by autophagy, proteasome, and protein aggregation. By culturing Neuro2a and HEK293 cells with ALS exosomes, the study demonstrated significantly higher TDP-43 cytoplasmic redistribution compared to cells treated with control exosomes, indicating the potential role of exosomes to act as seeds for prion propagation. However, the team also finds that inhibition of exosome secretion via GW4869 or RAB27A-led inactivation of neutral sphingomyelinase 2, a prominent mediator of exosome secretion in neurons, promoted TDP-43 aggregation in Neuro2a cells and led to worsened disease phenotypes in TDP-43^A315T^ mutant transgenic mice (Iguchi et al., 2016). These results suggest that exosomes not only function to propagate but can also act to eliminate TDP-43 aggregates in a constructive way, which complicates therapeutic strategies based simply on exosome inhibition. To investigate how TDP-43 is propagated through exosomes, Ding et al. (2015) treated human glioma U251 cells with ALS CSF, and found increased apoptosis, autophagy, TDP-43 aggregation as well as elevated number of exosomes and tunneling nanotubes (TNTs)-like structures. Noted, TNTs also function to transfer proteins and biomolecules between adjacent cells.

In addition to the most commonly found SOD1 and TDP-43 proteins, comprehensive proteomic analyses of exosomes have also found a number of less studied proteins associated with ALS including FUS mutations, aberrant hexanucleotide repeat expansions in *C9orf72*, and inflammation-related factors Interleukin-6 (IL-6) (Kamelgarn et al., 2016; Westergard et al., 2016; Chen B. Y. et al., 2019). In a study led by Hayashi et al., proteomic analysis of CSF exosomes revealed that levels of 14 different proteins were significantly changed between sALS patients and idiopathic normal-pressure hydrocephalus patients (Hayashi et al., 2020). Among these, nucleolar complex protein 2 homolog, a novel INHAT repressor, showed the highest level of elevation (Hayashi et al., 2020). In addition, Vassileff et al. (2020) reported that the expression of stress granule proteins STAU1 and DHX30 were significantly increased in ALS patient motor cortex brain tissues compared to neurological controls. Moreover, through liquid chromatography-tandem mass spectrometry, Thompson et al. (2020) reported the downregulation of Bleomycin hydrolase (BLMH) protein in ALS patient (*n* = 12) CSF compared to non-related healthy controls (*n* = 5). BLMH has been linked to AD and is involved in various cellular processes including brain homeostasis, energy metabolism, and synaptic plasticity. Downregulation of BLMH is associated with Hcy-thiolactone accumulation-induced seizures (Suszyńska-Zajczyk et al., 2014). Non-proteomic studies such as RNA-Seq analyses have also been preformed to predict ALS-associated proteins. For example, Swindell et al. (2019) demonstrated that blood exosomes of ALS patients show high EEF1A1, RPL13A, RPS6 levels. Otake et al. (2019) found 543 dysregulated mRNAs in ALS patient CSF exosomes compared to healthy controls. Among these, CUE domain-containing 2 (CUEDC2) protein was suggested as a potential ALS biomarker. Interestingly, CUEDC2 has been evidenced to play an important role in cerebral ischemia (Huang et al., 2021).

Since exosomes can readily cross the BBB, they may be probable candidates for non-viral vectors for target protein therapy. For example, exosomes loaded with siRNA targeting beta-secretase1 (BACE1), prominently found in AD pathogenesis, have been demonstrated to enter neurons, oligodendrocytes, and microglia (Alvarez-Erviti et al., 2011). Most important, this strategy effectively reduced BACE1 mRNA and protein by 60 and 62%, respectively. Overall, the studies reviewed in this section all point to the ability of exosomes to act as efficient carriers and propagators of proteins associated with ALS, and may help to explain how the disease is progressively spread throughout the body even when there is no underlying genetic cause.

## Exosomal miRNA in ALS

Although a significant amount of research has centered around misfolded proteins carried by exosomes, other constituents including extracellular RNAs have also garnered great interest in either detecting or perpetuating ALS progression. Extracellular RNAs such as messenger RNA (mRNA), microRNA (miRNA), and circular RNA (CirRNA) are secreted by extracellular vesicles and RNA binding proteins (RBP) (Kim et al., 2017). RNA contents inside the double-membrane exosomes are protected from ribonucleases found in biofluids, thus remain highly stable (Cheng et al., 2014a; Bonafede and Mariotti, 2017; Katsu et al., 2019; Wang and Zhang, 2020). Recently, aberrant expression of non-coding RNAs, particularly miRNAs, have been implicated in the pathogenesis of ALS. MiRNAs are highly conserved single-stranded RNAs comprised of around 19–24 nucleotides. They function by binding to either the 3′-untranslated region (UTR) or open reading frame (ORF) region of target mRNAs to mediate post-transcriptional gene silencing (Bartel, 2004; Chen and Rajewsky, 2007). In fact, evidence suggests that aberrant miRNA expression alone is capable of reprogramming neuron cell identity (Selbach et al., 2008; Yoo et al., 2011; Pomper et al., 2020). Changes in miRNA levels are stimulated under stressful conditions such as hypoxia and have been detected to regulate key proteins involved in ALS pathogenesis (Hosaka et al., 2019). In ALS patients, aberrant serum levels of miR1234-3p and miR-1825, as well as plasma levels of miR-130a-3p, miR-151b, and miR-221-3p have been correlated with sALS progression (Freischmidt et al., 2015; Liguori et al., 2018; Wang and Zhang, 2020). In addition, a study conducted by Freischmidt et al. (2013) discovered that TDP-43 protein binding miRNAs such as miR-132-5p, miR-132-3p, miR-143-5p, miR-143-3p, and miR-574-3p were significantly dysregulated in ALS patients and immortalized lymphoblast cell lines (LCLs). Despite multiple studies on general miRNA profiles, exosomal miRNAs, which may have more specific functions in mediating cell-cell communication in ALS, is a less studied area. In consideration to the critical regulating power of miRNAs in the CNS, analysis of exosomal miRNA contents in circulating body fluids such as blood, urine, and CSF can be a promising tool for understanding ALS onset and progression.

A study led by Xu et al. enlisted 10 ALS patients and 20 healthy controls to investigate a single miRNA (miR-27a03p) through quantitative real-time PCR (qRT-PCR) using serum-derived exosomes. They reportedly found a reduction in exosomal miR-27a-3p in ALS patients compared to healthy controls (Xu et al., 2018). Noted, this research group had previously found that exosomes carrying miR-27a-3p promoted osteoblast mineralization, which may be involved in ALS-related muscle-bone degeneration (Xu et al., 2018). Interestingly, miR-206 have also been implicated in muscle reinnervation in SOD1G93A mice, a transgenic mouse model with similar phenotypes as human ALS patients (Williams et al., 2009). In other studies that employed the SOD1G93A mouse model, aberrant expression of miR-124, miR125b, and miR-155 have been found to contribute to neuronal inflammation (Koval et al., 2013; Butovsky et al., 2015; Parisi et al., 2016; Yelick et al., 2020). In particular, miR-124, one of the most abundant miRNAs in neuronal exosomes, is associated with neuronal cell differentiation during early embryogenesis (Pinto et al., 2017; Yelick et al., 2020). Previous research led by Morel et al. (2013) has also indicated that exosomal miR-124a can stimulate glutamate transporter GLT1 expression in astrocytes. In addition, exosomes from NSC-34 cells stably transfected with mSOD1 showed higher miR-124 levels compared to with wt SOD1-transfected cells (Pinto et al., 2017). More important, when exosomes isolated from mSOD1 NSC-34 cells were co-incubated with N9 microglia cells, the phagocytic ability of N9 cells was reduced, and that the nuclear factor κB signaling pathway was activated to increase the release of various cytokines including IL-1β, TNF-α, MHC-II, and iNOS (Pinto et al., 2017). The seemingly important role of miR-124 in the CNS drove researchers to further investigate its potential role in ALS pathogenesis. For example, Yelick et al. (2020) examined CSF-derived exosomes from ALS patients (*n* = 14), neurological disease controls (*n* = 9), and healthy controls (*n* = 9) using qPCR, and found that miR-124-3p levels positively correlated with disease severity in male ALS patients. The study also found increased miR-124-3p levels in the spinal motor neuron-derived exosomes of SOD1G93A mice.

In addition to single exosomal miRNA studies, researchers have also broadly investigated changes in the miRNA profile of ALS patients. However, instead of exosomes, these studies elected to study miRNA profiles isolated from all EVs (comprising exosomes). In a study with 5 ALS patients and 5 healthy controls, Katsu et al. (2019) analyzed the miRNA profiles of neuron-derived EVs from plasma samples using microarray. The study reported a total of 30 differentially regulated miRNAs (13 up-regulated and 17 down-regulated) in ALS patients compared to the controls. Gene ontology analysis suggested that these 30 differentially expressed miRNAs were involved in synaptic vesicle-related pathways, including synaptic vesicle docking and exocytosis, regulation of neurotransmitter secretion, and synaptic vesicle cycle. When comparing the miRNA expression profiles between plasma neuron-derived samples and formalin-fixed paraffin-embedded motor cortex samples from ALS patients, four out of the 30 dysregulated miRNAs were found to be similarly regulated. Specifically, miR-24-3p was up-regulated, while miR-1268a, miR-3911, and miR-4646-5p were down-regulated, and all are capable of targeting ATX1B, RAB3B, and UNC13A genes (Katsu et al., 2019). Notably, previous genome-wide association studies have reported the positive relationship between UNC13A expression and increased likelihood of sALS onset (van Es et al., 2009; Ahmeti et al., 2013; Diekstra et al., 2014). In a study led by Saucier et al., next-generation sequencing (NGS) was employed to examine differentially expressed miRNAs in plasma-derived EVs (Saucier et al., 2019). Specifically, a total of five up-regulated and 22 down-regulated miRNAs were identified in ALS patients (*n* = 14) compared to healthy controls (*n* = 8), four of which were functionally involved in transcriptional regulation and protein ubiquitination, and may be of high relevance to ALS (miR-9-5p, miR-183-5p, miR-338-3p, and miR-1246). Moreover, miR-15a-5p and miR-193-5p were recognized for their diagnostic potential and disability progression (Saucier et al., 2019). More recently, using plasma-derived EVs from ALS patients (*n* = 10) and controls (*n* = 10), Banack et al. (2020) identified 101 differentially expressed miRNA by NGS, though none were sensitive enough to function as biomarkers for ALS.

As consolidated in Table 1, various extracellular miRNAs have been linked to ALS onset and progression; however, the lack of overlapping results among these studies undermine the diagnostic and mechanistic values of these findings. Part of the reason for such inconsistency may be due to a lack of standard detection and analysis methods as well as the varied biological samples employed. For instance, in human plasma, miRNA makes up 76.2% of total nucleic acid content compared to only 35% found in human urine. Moreover, different methods of detection can lead to differential results even employing the same type of biological fluid. For example, in human urine, a study using total RNA-seq failed to detect any miRNA content, while it was found highly abundant (35%) when using small RNA-seq (Cheng et al., 2014b; Miranda et al., 2014; Turchinovich et al., 2019).

**TABLE 1:** Differentially expressed exosomal miRNAs in ALS.

Exosomal miRNA	Expression Level	Sample Type	Sample Size	Exosome Isolation Method	miRNA Detection Method	References
miR-124	Up	NSC-34 Cells	NA	UC	qRT-PCR	Pinto et al., 2017
miR-124a	Up	Astrocyte	NA	UC	qRT-PCR	Morel et al., 2013
miR-124-3p	Up	CSF	4 patients; 9 disease controls; 9 healthy controls	Total Exosome Isolation Reagent (Thermo Fisher)	qRT-PCR	Yelick et al., 2020
miR-4736	Up	Plasma	5 patients; 5 healthy controls	Immuno-affinity	3D-Gene chip	Katsu et al., 2019
miR-4700-5p	Up	Plasma	5 patients; 5 healthy controls	Immuno-affinity	3D-Gene chip	Katsu et al., 2019
miR-1207-5p	Up	Plasma	5 patients; 5 healthy controls	Immuno-affinity	3D-Gene chip	Katsu et al., 2019
miR-4739	Up	Plasma	5 patients; 5 healthy controls	Immuno-affinity	3D-Gene chip	Katsu et al., 2019
miR-4505	Up	Plasma	5 patients; 5 healthy controls	Immuno-affinity	3D-Gene chip	Katsu et al., 2019
miR-24-3p	Up	Plasma	5 patients; 5 healthy controls	Immuno-affinity	3D-Gene chip	Katsu et al., 2019
miR-149-3p	Up	Plasma	5 patients; 5 healthy controls	Immuno-affinity	3D-Gene chip	Katsu et al., 2019
miR-4484	Up	Plasma	5 patients; 5 healthy controls	Immuno-affinity	3D-Gene chip	Katsu et al., 2019
miR-4688	Up	Plasma	5 patients; 5 healthy controls	Immuno-affinity	3D-Gene chip	Katsu et al., 2019
miR-4298	Up	Plasma	5 patients; 5 healthy controls	Immuno-affinity	3D-Gene chip	Katsu et al., 2019
miR-939-5p	Up	Plasma	5 patients; 5 healthy controls	Immuno-affinity	3D-Gene chip	Katsu et al., 2019
miR-371a-5p	Up	Plasma	5 patients; 5 healthy controls	Immuno-affinity	3D-Gene chip	Katsu et al., 2019
miR-3619-3p	Up	Plasma	5 patients; 5 healthy controls	Immuno-affinity	3D-Gene chip	Katsu et al., 2019
miR-4454	Up	Plasma	14 patients; 8 healthy controls	Vn peptides	NGS	Saucier et al., 2019
miR-9-1-5p	Up	Plasma	14 patients; 8 healthy controls	Vn peptides	NGS	Saucier et al., 2019
miR-9-3-5p	Up	Plasma	14 patients; 8 healthy controls	Vn peptides	NGS	Saucier et al., 2019
miR-338-3p	Up	Plasma	14 patients; 8 healthy controls	Vn peptides	NGS	Saucier et al., 2019
miR-9-2-5p	Up	Plasma	14 patients; 8 healthy controls	Vn peptides	NGS	Saucier et al., 2019
miR-100-5p	Up	Plasma	14 patients; 8 healthy controls	Vn peptides	NGS	Saucier et al., 2019
miR-7977	Up	Plasma	14 patients; 8 healthy controls	Vn peptides	NGS	Saucier et al., 2019
miR-1246	Up	Plasma	14 patients; 8 healthy controls	Vn peptides	NGS	Saucier et al., 2019
miR-664a-5p	Up	Plasma	14 patients; 8 healthy controls	Vn peptides	NGS	Saucier et al., 2019
miR-7641-1	Up	Plasma	14 patients; 8 healthy controls	Vn peptides	NGS	Saucier et al., 2019
miR-1290	Up	Plasma	14 patients; 8 healthy controls	Vn peptides	NGS	Saucier et al., 2019
miR-4286	Up	Plasma	14 patients; 8 healthy controls	Vn peptides	NGS	Saucier et al., 2019
miR-181b-1-5p	Up	Plasma	14 patients; 8 healthy controls	Vn peptides	NGS	Saucier et al., 2019
miR-1260b	Up	Plasma	14 patients; 8 healthy controls	Vn peptides	NGS	Saucier et al., 2019
miR-181b-2-5p	Up	Plasma	14 patients; 8 healthy controls	Vn peptides	NGS	Saucier et al., 2019
miR-127-3p	Up	Plasma	14 patients; 8 healthy controls	Vn peptides	NGS	Saucier et al., 2019
miR-181a-2-5p	Up	Plasma	14 patients; 8 healthy controls	Vn peptides	NGS	Saucier et al., 2019
miR-181a-1-5p	Up	Plasma	14 patients; 8 healthy controls	Vn peptides	NGS	Saucier et al., 2019
miR-199a-2-3p	Up	Plasma	14 patients; 8 healthy controls	Vn peptides	NGS	Saucier et al., 2019
miR-199a-1-3p	Up	Plasma	14 patients; 8 healthy controls	Vn peptides	NGS	Saucier et al., 2019
miR-27a-3p	Down	Serum	10 patients; 20 controls	ExoQuick and exoEasy Maxi Kit	qRT-PCR	Xu et al., 2018
miR-1268a	Down	Plasma	5 patients; 5 healthy controls	Immuno-affinity	3D-Gene chip	Katsu et al., 2019
miR-2861	Down	Plasma	5 patients; 5 healthy controls	Immuno-affinity	3D-Gene chip	Katsu et al., 2019
miR-4508	Down	Plasma	5 patients; 5 healthy controls	Immuno-affinity	3D-Gene chip	Katsu et al., 2019
miR-4507	Down	Plasma	5 patients; 5 healthy controls	Immuno-affinity	3D-Gene chip	Katsu et al., 2019
miR-3176	Down	Plasma	5 patients; 5 healthy controls	Immuno-affinity	3D-Gene chip	Katsu et al., 2019
miR-4745-5p	Down	Plasma	5 patients; 5 healthy controls	Immuno-affinity	3D-Gene chip	Katsu et al., 2019
miR-3911	Down	Plasma	5 patients; 5 healthy controls	Immuno-affinity	3D-Gene chip	Katsu et al., 2019
miR-3605-5p	Down	Plasma	5 patients; 5 healthy controls	Immuno-affinity	3D-Gene chip	Katsu et al., 2019
miR-150-3p	Down	Plasma	5 patients; 5 healthy controls	Immuno-affinity	3D-Gene chip	Katsu et al., 2019
miR-3940-3p	Down	Plasma	5 patients; 5 healthy controls	Immuno-affinity	3D-Gene chip	Katsu et al., 2019
miR-4646-5p	Down	Plasma	5 patients; 5 healthy controls	Immuno-affinity	3D-Gene chip	Katsu et al., 2019
miR4687-5p	Down	Plasma	5 patients; 5 healthy controls	Immuno-affinity	3D-Gene chip	Katsu et al., 2019
miR-4788	Down	Plasma	5 patients; 5 healthy controls	Immuno-affinity	3D-Gene chip	Katsu et al., 2019
miR-4674	Down	Plasma	5 patients; 5 healthy controls	Immuno-affinity	3D-Gene chip	Katsu et al., 2019
miR-1913	Down	Plasma	5 patients; 5 healthy controls	Immuno-affinity	3D-Gene chip	Katsu et al., 2019
miR-634	Down	Plasma	5 patients; 5 healthy controls	Immuno-affinity	3D-Gene chip	Katsu et al., 2019
miR-3177-3p	Down	Plasma	5 patients; 5 healthy controls	Immuno-affinity	3D-Gene chip	Katsu et al., 2019
miR-532-3p	Down	Plasma	14 patients; 8 healthy controls	Vn peptides	NGS	Saucier et al., 2019
miR-144-3p	Down	Plasma	14 patients; 8 healthy controls	Vn peptides	NGS	Saucier et al., 2019
miR-15a-5p	Down	Plasma	14 patients; 8 healthy controls	Vn peptides	NGS	Saucier et al., 2019
miR-363-3p	Down	Plasma	14 patients; 8 healthy controls	Vn peptides	NGS	Saucier et al., 2019
miR-183-5p	Down	Plasma	14 patients; 8 healthy controls	Vn peptides	NGS	Saucier et al., 2019
101 significantly differentially expressed miRNA	−	Plasma	10 patients; 10 healthy controls	ExoQuick (SBI)	NGS and qPCR	Banack et al., 2020

## Exosome Isolation Techniques and Limitations

**FIGURE 2:**
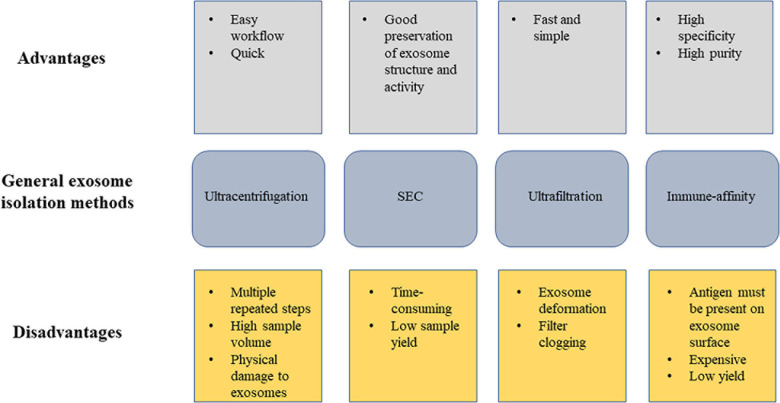
Illustration of exosome isolation strategies in current ALS studies. This figure depicts the advantages and disadvantages of the general exosome isolation methods used in currently available ALS studies.

Exosomes exist in all body fluids, and thus serve as an optimal candidate for non-invasive diagnostic biomarker. However, a major challenge for current exosome research is the lack of a standard isolation technique, part of which is due to the heterogenous nature of exosomes. While the introduction of new methodologies have made exosome studies possible for clinical diagnosis and treatment, various issues including low yield, non-reproducibility, contamination, and time-consumption continue to challenge researchers in this field. Current methods for exosomal isolation include sucrose density gradient ultracentrifugation, ultrafiltration (UF), size-exclusion liquid chromatography (SEC), paper-based immunoaffinity system, microfluidic system, and precipitation using immunoaffinity beads, heparin affinity or polyethylene glycol precipitation, also shown in Figure 2 (Batrakova and Kim, 2015; Chen et al., 2015; Peterson et al., 2015; Cho et al., 2016; Li et al., 2017; Momen-Heravi, 2017; Chen B. Y. et al., 2019). A search through currently available ALS studies show a variety of methods used for exosome extraction, including sucrose gradient centrifugation, immune-affinity, SEC, isolation kits, and UC (Table 2). The most commonly used method is sucrose density gradient ultracentrifugation, which involves serial ultracentrifugation followed by a 30% sucrose cushion for improved purification. This method is simple to navigate, but is time-consuming and less efficient in eliminating impurities from the parent liquid. Most common protein contaminants from parent liquid include albumin, matrix metalloproteases, and immunoglobulins, which are found highly abundant in body fluids (Mincheva-Nilsson et al., 2016; Ludwig et al., 2019; Takov et al., 2019). While repeated centrifugation can be done to remove non-EV particles, there are major drawbacks including low RNA yield and physical damage to the vesicles. And although ultracentrifugation has proven to be effective in elucidating exosomes from single cell lines since the starting sample load can always be increased, working with limited and more complicated biological fluids such as plasma, CSF, and serum remains a major challenge. The quality of the isolated exosomes is highly dependent on the isolation technique, and will have significant impact on results analysis and interpretation. Multiple studies have also indicated that variation in method adopted will yield different exosome profiles (Rekker et al., 2014; Tang et al., 2017; Ding et al., 2018).

**TABLE 2:** Altered levels of exosomal proteins in ALS.

Protein	Sample Type	Sample Size	Exosome Isolation Method	Detection method	References
SOD1	NSC-34 cells	NA	Sucrose gradient centrifugation	Western blot: CD9	Gomes et al., 2007
SOD1	Astrocytes	NA	UC	Electron Microscopy/Western blot: flotillin-1	Basso et al., 2013
SOD1	NSC-34 cells	NA	UC	Electron Microscopy	Grad et al., 2014
SOD1	Microglia	NA	UC	Atomic force microscopy (AFM)	Massenzio et al., 2018
SOD1	Mouse/human brain and spinal cord tissues	5 patients; 4 controls	Sucrose gradient centrifugation	Western blot: SOD1, PrP, actin, GRP78/Bip, CD9	Silverman et al., 2019
TDP-43	SH-SY5Y cells	NA	ExoQuick-TC kit (SBI)	CD63	Nonaka et al., 2013
TDP-43	CSF	18 patients; 8 ALS plus FTD patients; 15 controls	UC	Western blot: Flotillin 1	Ding et al., 2015
TDP-43	Neuro2a cells and primary neurons brain tissues mouse serum	3 patients; 3 controls	UC	Electron microscopy Western blot: CD63 and ALIX	Iguchi et al., 2016
TDP-43	Serum	18 patients	Immuno-affinity	Transmission Electron Microscopy	Chen et al., 2020
FUS	SH-SY5Y and N2A cells	NA	UC	Western blot: ALIX, TSG101 and CD63	Kamelgarn et al., 2016
DPR	NSC-34 cells	NA	Total Exosome Isolation kit (Thermo Fisher)	Western blot: flotillin, TSG101, and CD63	Westergard et al., 2016
NOC2L	CSF	3 patients; 3 controls	SEC	Western blot analysis: CD9, CD81 Electron microscopy: CD81, CD9, and NIR	Hayashi et al., 2020
PDCD6IP	CSF	3 patients; 3 controls	SEC	Western blot analysis: CD9, CD81 Electron microscopy: CD81, CD9, and NIR	Hayashi et al., 2020
VCAN	CSF	3 patients; 3 controls	SEC	Western blot analysis: CD9, CD81 Electron microscopy: CD81, CD9, and NIR	Hayashi et al., 2020
SERPINA3	CSF	3 patients; 3 controls	SEC	Western blot analysis: CD9, CD81 Electron microscopy: CD81, CD9, and NIR	Hayashi et al., 2020
PTPRZ1	CSF	3 patients; 3 controls	SEC	Western blot analysis: CD9, CD81 Electron microscopy: CD81, CD9, and NIR	Hayashi et al., 2020
C1QC	CSF	3 patients; 3 controls	SEC	Western blot analysis: CD9, CD81 Electron microscopy: CD81, CD9, and NIR	Hayashi et al., 2020
CCDC19	CSF	3 patients; 3 controls	SEC	Western blot analysis: CD9, CD81 Electron microscopy: CD81, CD9, and NIR	Hayashi et al., 2020
MYL6B	CSF	3 patients; 3 controls	SEC	Western blot analysis: CD9, CD81 Electron microscopy: CD81, CD9, and NIR	Hayashi et al., 2020
MARCO	CSF	3 patients; 3 controls	SEC	Western blot analysis: CD9, CD81 Electron microscopy: CD81, CD9, and NIR	Hayashi et al., 2020
FCGBP	CSF	3 patients; 3 controls	SEC	Western blot analysis: CD9, CD81 Electron microscopy: CD81, CD9, and NIR	Hayashi et al., 2020
FOLR1	CSF	3 patients; 3 controls	SEC	Western blot analysis: CD9, CD81 Electron microscopy: CD81, CD9, and NIR	Hayashi et al., 2020
RELN	CSF	3 patients; 3 controls	SEC	Western blot analysis: CD9, CD81 Electron microscopy: CD81, CD9, and NIR	Hayashi et al., 2020
CFB	CSF	3 patients; 3 controls	SEC	Western blot analysis: CD9, CD81 Electron microscopy: CD81, CD9, and NIR	Hayashi et al., 2020
CHMP4A	CSF	3 patients; 3 controls	SEC	Western blot analysis: CD9, CD81 Electron microscopy: CD81, CD9, and NIR	Hayashi et al., 2020
Interleukin-6	plasma	40 patients; 39 controls	Immuno-affinity	Western blot: CD63 Transmission electron microscope	Chen Y. et al., 2019
STAU1	Motor cortex	10 patients; 5 controls	Sucrose gradient centrifugation	Western blot: flotillin-1, TSG101 and syntenin Transmission electron microscopy (TEM)	Vassileff et al., 2020
DHX30	Motor cortex	10 patients; 5 controls	Sucrose gradient centrifugation	Western blot: flotillin-1, TSG101 and syntenin Transmission electron microscopy (TEM)	Vassileff et al., 2020
189 proteins	ASC cells	NA	PureExo^®^ Exosome isolation kit	Transmission electron microscopy (TEM) Western blot: HSP70, CD9, and CD81	Bonafede et al., 2019

Currently, there is a limited number of ALS studies based on exosome isolation. Notably, there are 16 articles on exosomal protein and only 7 on exosomal miRNA. A quick look at Tables 1, 2 indicates that these studies are highly variable in terms of not only exosome isolation technique, but also sample type and detection method. For example, Katsu et al. (2019) conducted a 3D-gene chip analysis based exosomes isolated from plasma through immune-affinity and identified 30 differentially regulated miRNAs. On the other hand, Saucier et al. (2019) used Vn peptides to isolate plasma exosomes for NGS, and found 25 differentially expressed miRNAs, none of which overlapped with findings from Katsu et al. Moreover, Banack et al. (2020) found 101 differentially expressed miRNAs based on NGS and exosomes isolated from plasma using ExoQuick isolation kit. Interestingly, while both Banack et al. and Saucier et al. used NGS-based detection method, the large variation in miRNA profile may be a result of different exosome isolation techniques performed. Considering inherent exosome heterogeneity, exosomes isolated using method A may be an entirely separate subpopulation compared to those extracted using method B. Nonetheless, the diverse employment of isolation and detection methods coupled with only a few studies may hinder the precision and significance these findings. To ensure data reliability and reproducibility, future studies should aim to find consistent methodologies for both exosome isolation and detection.

## Conclusion

Remarkable progress has been made since exosomes were first discovered almost 40 years ago, catapulting the field to the forefront of neurobiology, cancer biology, immunology, clinical diagnosis, and drug delivery. The study of exosomes in ALS pathogenesis is still a relatively new field. Though the ability for them to act as cargos and propagators of misfolded proteins and functional miRNAs have been clearly evidenced in literature, less is known about the underlying mechanisms. For one, considering the highly heterogenous nature of exosomes, it is unclear whether disease-related proteins are actively sorted into these vesicles or simply through random bulk cytoplasm and membrane inclusion.

The cargo sorting mechanism is also essential for understanding the potential for exosomes to act as therapeutic agents. On account of the ability for exosomes to carry a wide range of biomolecules, development of exosomes as models for drug delivery will certainly expand its utility and applicability. For one, exosomes can be powerful therapeutic tools for neurodegenerative diseases due to their innate stability, high affinity, low toxicity, and ability to cross the BBB. Moreover, because exosomes can be derived from the patient’s own cells, there is little chance for foreign rejection by the immune system. There are a few studies that have incorporated exosomes as therapeutic agents for ALS. Stem cells have the tremendous ability to self-renew and differentiate, which are important characteristics for damage repair and regeneration. Several studies have reported that exosomes isolated from adipose-derived stem cells (ASCs) can promote neuronal protection and regeneration. As shown by Lee et al., G93A NSCs treated with exosomes isolated from ASCss demonstrated reduced cytosolic SOD1 levels and normalized levels of PGC-1 α as well as phospho-CREB/CREB ratio (Lee et al., 2016). Bonafede et al. (2016) also showed that exosomes from adipose-derived stromal cells have the ability to protect NSC-34 cells from oxidative stress. Similarly, mutated SOD1(G93A) NSC-34 cells treated with ASC-derived exosomes have also been shown to improve mitochondrial complex I activity and mitochondrial membrane potential (Calabria et al., 2019). *In vivo*, Bonadede et al. showed that administration of exosomes isolated from ASCs can result in exosome deposition in lesioned areas of the brain, lumbar motoneuron protection, and improvement of motor performance in SOD1(G93A) mice. However, to date, research incorporating the use of exosomes as therapeutic tools for ALS is very limited. Most of the studies, as mentioned above, rely on the innate regenerative functions of stem cell-derived exosomes. However, there is a lack in testing exosomes as vehicles for drug and gene therapy for ALS. In other neurodegenerative diseases, exosomes have been used for siRNA delivery to deliberately target disease proteins, such as *BACE1* in Alzheimer’s disease (Alvarez-Erviti et al., 2011). In addition, in a Parkinson’s disease mouse model, intranasal administration of catalase-loaded exosomes provided significant neuroprotective effects (Haney et al., 2015). Future studies should explore the potential for exosomal gene delivery for ALS treatment, both *in vitro* and *in vivo*.

Apart from acting as vehicles for therapeutic drugs, exosomes can also act as protective coats for therapeutic nanoparticles (NPs). NPs are known to transport active biomolecules across membranes with great stability and for a comparatively longer amount of time compared to traditional drug delivery systems. In addition, engineered NPs can be designed to target cells with high specificity. However, unlike exosomes, NPs are recognized as foreign bodies and often elicit immunological responses. Thus, some have suggested that conjugation of NPs and exosomes may be a perfect match to achieve safe delivery of therapeutic agents. However, it is currently unknown whether or not exosomes can disrupt target specificity of NPs. For example, coating NPs with exosomes from stem cells that are larger in size may target cells differently compared to NPs coated with exosomes from blood cells that are smaller in size. In consideration to this, future studies should examine the effect of exosome heterogeneity on potential off-target effects of NP-exosome drug delivery systems.

These efficient nanoparticles are valuable candidates for studying ALS pathogenesis in that they can act as both biomarkers for disease progression as well as efficient drug delivery vehicles for therapeutic purposes. Despite much progress in this field, there still exist many obstacles, including the basic challenges such as the lack of standardized exosome isolation and detection techniques, creating inconsistencies between different studies. Moreover, studies in the field have several limitations. For one, currently available proteomic analyses and miRNA screenings only reveal alterations in potential disease-related protein and miRNA levels. However, like in many diseases such as cancer, it is widely known that alteration in one gene, miRNA, or protein is not enough to drive the disease forward. The driver gene, once activated, can elicit an array of downstream gene dysregulation. Similarly, one would assume that ALS disease initiation and progression is more complicated than alterations in a few critical proteins and miRNAs. A comprehensive look through current literature shows that there are numerous dysregulated miRNAs and proteins found associated with ALS. But the critical question to ask is whether they are all important for ALS disease progression. Thus, signaling pathway and network analyses involving protein-protein or protein-RNA interactions should be important next steps toward understanding the underlying pathogenic mechanisms of ALS.

## Author Contributions

QC and JD conceived the topic and wrote the manuscript. QC, TW, and PW led in the writing. RJ and RZ contributed to literature review. All authors contributed to the article and approved the submitted version.

## Conflict of Interest

The authors declare that the research was conducted in the absence of any commercial or financial relationships that could be construed as a potential conflict of interest.

## Publisher’s Note

All claims expressed in this article are solely those of the authors and do not necessarily represent those of their affiliated organizations, or those of the publisher, the editors and the reviewers. Any product that may be evaluated in this article, or claim that may be made by its manufacturer, is not guaranteed or endorsed by the publisher.

## Abbreviations

α-syn: α-synuclein
A β: amyloid beta
ABs: apoptotic bodies
AD: Alzheimer’s disease
ALS: amyotrophic lateral sclerosis
Arc: activity-regulated-cytoskeleton-associated protein
Atg5: autophagy-related 5
BBB: blood brain barrier
CNS: central nervous system
CSF: cerebrospinal fluid
DPRs: dipeptide-repeat proteins
DPYL: dihydropyrimidinase-related proteins
ECM: extracellular matrix
ESCRT: endosomal sorting complex required for transport
EVs: extracellular vesicles
fALS: familial ALS
FUS: first apoptosis signal
hnRNP: heterogenous nuclear ribonucleoprotein
MCT1: monocarboxylate transporter
miRNA: microRNA
MN: motor neuron
mRNA: messenger RNA
MSCs: mesenchymal stem cells
MVs: microvesicles
MVBs: multi-vesticular bodies
ND: neurodegenerative diseases
NGS: next generation sequencing
ORF: open reading frame
PD: Parkinson’s disease
qRT-PCR: quantitative real-time PCR
RBP: RNA binding proteins
sALS: sporatic ALS
SEC: size-exclusion liquid chromatography
SOD1: superoxide dismutase 1
TDP-43: TAR DNA binding protein 43
TNTs: tunneling nanotubes
UF: ultrafiltration
UTR: untranslated region
WT: wild-type.
